# Nutritional adequacy of meals and commissary items provided to individuals incarcerated in a southwest, rural county jail in the United States

**DOI:** 10.1186/s40795-022-00593-w

**Published:** 2022-09-03

**Authors:** Nanette V. Lopez, Ary Spilkin, Julianne Brauer, Rachelle Phillips, Bonnie Kuss, Gabrielle Delio, Ricky Camplain

**Affiliations:** 1 Department of Health Sciences, Northern Arizona University, 1100 South Beaver Street Box 15095, Flagstaff, AZ 86011 USA; 2 Center for Health Equity Research, Northern Arizona University, Flagstaff, AZ USA

**Keywords:** Incarceration, Jail menu, Nutrition

## Abstract

**Background:**

Poor diet may contribute to deleterious chronic health among individuals incarcerated. Yet, limited research has evaluated the nutritional content of menus and commissary items provided in jails. Thus, this study assessed the macronutrient distribution, caloric composition, and diet quality of the seven-day cycle menu and commissary items provided in a southwest, rural county jail in the United States.

**Methods:**

Daily and mean availability of calories and macronutrients for the seven-day cycle menu and commissary items were estimated using NutritionCalc Plus®. Diet quality (i.e., Healthy Eating Index-2015 [HEI-2015]) was assessed. Macronutrients and calories were compared to the Acceptable Macronutrient Distribution Range (AMDR) and the 2020–2025 Dietary Guidelines for Americans (DGA). Protein and carbohydrate were compared to the Dietary Reference Intake (DRI). HEI-2015 was compared to the average U.S. diet.

**Results:**

Daily caloric provisions exceeded DGA recommendations. Daily available (16.2%-25.2% kcal/day) and mean protein met the AMDR recommendations, yet exceeded the DRI. Mean protein with commissary packs exceeded the AMDR recommendations and DRI. Daily available carbohydrate met AMDR recommendations for all but two days of the seven-day cycle menu, which exceeded recommendations (52.5%-66.4% kcal/day). Mean carbohydrate met the AMDR recommendations and exceeded the DRI, and with the commissary packs, exceeded the AMDR recommendations and DRI. Daily available total fat for the seven-day cycle menu (79.5–146.7 g), mean total fat alone and with the commissary packs exceeded AMDR recommendations. Daily available saturated fat for the seven-day cycle menu (16.7–47.7 g) exceeded AMDR recommendations for all but one day of the seven-day cycle menu, while mean saturated fat alone and with the commissary packs exceeded AMDR recommendations. Daily available added sugars for the seven-day cycle menu (8.4–14.2 g), mean added sugars alone and with the commissary packs all met AMDR recommendations. HEI-2015 scores for the seven-day cycle menu ranged from 49.3–74.5 (mean = 62.2, SD = 9.4), and increased with the commissary packs.

**Conclusions:**

Exceeding caloric and saturated fat recommendations may contribute to weight gain, regardless of high diet quality. Increasing nutrient-dense foods available in jail may reduce chronic disease among incarcerated populations.

**Supplementary Information:**

The online version contains supplementary material available at 10.1186/s40795-022-00593-w.

## Introduction

Over 740,000 U.S. adults are incarcerated in county jails [1], which house individuals awaiting adjudication or serving sentences < 1 year. Individuals incarcerated experience numerous health inequities, including higher rates of chronic disease, such as hypertension, type 2 diabetes, and heart disease [2, 3]. Although the average length of a jail stay is 8–12 days [4, 5], individuals incarcerated may remain incarcerated for 8 years [6]. Additionally, recidivism rates for individuals incarcerated in Arizona are among the highest in the country at 42.4% [6]. Thus, recurrent jail stays and potential prison time place individuals incarcerated in a position to continual exposure to unhealthy environments impacting acute and chronic health.

Poor diet quality may potentially contribute to deleterious chronic health among individuals incarcerated. Staff create menus in jails, allowing individuals incarcerated little to no autonomy over food choices. Creation of meals often prioritizes financial constraints due to the use of the public’s money funding foodservice[7] rather than meeting nutritional recommendations [8], including the use of lower cost, high-calorie foods that are less nutritionally adequate. In 2019, Arizona correctional facilities spent, on average, $3.22 per meal to feed an individual who was incarcerated [9]. For the menu in the current study, the average daily cost was $2.58, or $0.86 per meal.

Guidelines for nutritional intake are based on Dietary Reference Intakes (DRIs), a set of nutritional reference values for all nutrients set by the Food and Nutrition Board of the Institute of Medicine (IOM). The DRIs represent quantitative approximations of nutrient needs for the purposes of planning and assessing the diet of healthy people [10]. The DRIs include Recommended Dietary Allowances (RDAs) that provide the average daily dietary intake sufficient to meet the nutrient requirements of 97% of healthy people. In addition to the DRIs, the IOM also determined the Acceptable Macronutrient Distribution Range (AMDR) for protein (10–35% of energy), carbohydrate (45–65% of energy), and fat (20–35% of energy) [11]. While the AMDR provides guidance only for macronutrient needs for all active adults [11], the DRIs provide approximations for all daily dietary needs. There is no established DRI for total fats, saturated fats or added sugars [10].

In addition to the DRIs, the Dietary Guidelines for Americans (DGAs) provide calorie intake recommendations based upon physical activity level [10]. A recent study conducted among all individuals who are incarcerated in the county jail for which we analyzed the seven-day cycle menu indicated that 73% of those surveyed reported sometimes or never engaging in recreation-time physical activity [12]. Additionally, another study examining only women who are incarcerated at the same county jail found that among those who engaged in recreation-time activity, almost 60% were sedentary during the dedicated time [13]. Per the DGAs, total energy recommendations for sedentary adults are 2,400 kcal for men and 1,800 kcal for women, with a 2,000 kcal recommendation falling within this range [10].

Limited research has examined the nutritional content of menus in jails. Meals served in South Carolina facilities contained excess levels of the DRIs for cholesterol, sugar, and sodium while several nutrients did not meet the DRIs including carbohydrate, fiber, calcium, Vitamins D and E, magnesium, and potassium [14]. Additionally, a 28-day cycle menu in a Georgia county jail provided excess calories for women incarcerated, and excess cholesterol, saturated fat, and sodium for all individuals incarcerated. Servings of grains were overrepresented while fruits, vegetables, fiber, and dairy were underrepresented [15]. The combination of excess cholesterol, sugar, fats, and sodium paired with limited vitamins, fruits, vegetables, fiber, and dairy may establish a nutritionally vulnerable environment that contributes to poor health outcomes among individuals incarcerated compared to other populations.

One controllable aspect of the food environment is the commissary, a store within the jail where individuals incarcerated or their friends/family can purchase hygiene, food, and stationery items. While commissary foods can contribute to calorie and macronutrient intake, limited research has explored the relationship between commissary foods and dietary impact. Researchers previously determined an average of 1,000 kcal per day was purchased daily at a commissary in a women’s jail in Oregon [16] and another group found that commissary meals provided excess calories, sodium, and fat [17]. However, a recipe book was used to analyze meals commonly prepared using commissary items rather than evaluate the individual items [17]. No known study has assessed the nutritional impact of available commissary foods in addition to menu provisions within the jail.

Examining the nutritional content and dietary quality of the menu and commissary items can provide a better understanding of food available to incarcerated populations and help assess the risk of chronic conditions. Thus, the purpose of this study is threefold: 1) to describe the macronutrient distribution (e.g., carbohydrate, protein, total fat, saturated fat, added sugars) and caloric provisions of a seven-day cycle menu and commissary items that can be purchased at a detention center in a rural county in the southwestern United States, 2) examine the compliance of the seven-day cycle menu and commissary items with the Acceptable Macronutrient Distribution Ranges (AMDR) and the United States Department of Agriculture’s (USDA) 2020–2025 Dietary Guidelines for Americans (DGAs), and 3) determine diet quality of the seven-day cycle menu and commissary items as assessed by the Healthy Eating Index-2015 (HEI-2015).

## Methods

A rural county jail in the southwestern United States, housing 450 men and women daily, provided a seven-day cycle menu and list of commissary items in November 2019. The seven-day cycle menu is repeated weekly, with an optional weekly purchase of commissary items, including food packs comprising pre-packaged foods. The menu analyzed is a representation of the seven-day cycle menu offered throughout the year. Annual reviews of diets are conducted by a Registered Dietitian to determine nutritional content. Individuals incarcerated or their family/friends may purchase commissary items, provided by a third party vendor, totaling no more than $80.00 USD weekly. Northern Arizona University Institutional Review Board provided an exemption for the current study because human subjects were not involved as per U.S. Department of Health and Human Services guidelines (http://www.hhs.gov/ohrp/policy/checklists/decisioncharts.html#c1).

## Seven-day cycle menu data entry

Two researchers entered the seven-day cycle menu into a dietary assessment tool used to assess macronutrients (NutritionCalc® Plus version 5.0.19, McGraw-Hill Education, New York, NY, 2018) [18]. A reference male (36 years old, 5′10″ tall, weighing 200 lb/90.9 kg) and female (36 years old, 5′ 3″ tall, weighing 169 lb/76.8 kg) [19] were used to determine the DRIs for macronutrients in the seven-day cycle menu. A 36-year-old individual was chosen because the greatest numbers of individuals incarcerated are aged 36 to 40 years [20]. Weight data for the reference male and female were identified from 2015–2016 Centers for Disease Control and Prevention data detailing estimates of mean body weight for adult men and women aged 20 years and over [21]. Discrepancies in data entry were reviewed by another team member. Individual profiles were created for the menu provisions for each day of the week (Sunday-Saturday) and meals were entered as breakfast, lunch, and dinner. Each menu item was entered as if eaten in its entirety, using the exact gram or ounce amounts indicated and “USDA” whenever possible. If NutritionCalc® Plus lacked a food or recipe in the database, entries were modified to represent the nutritional value of the menu item. Fruits were inputted as “fresh” and beans and vegetables were inputted as “canned” (Supplementary Table 1).

The same two researchers entered the menu into the Automated Self-Administered 24-h Dietary Assessment Tool (ASA24®) [22], another dietary assessment tool used to assess the dietary quality of the menu. ASA24® was used to determine the overall Healthy Eating Index-2015 (HEI-2015) [23] score and to provide added sugars, measured in grams. Individual profiles and meal entries were completed identically as reported for NutritionCalc® Plus. Food items were converted into tablespoons and cups when appropriate.

### Commissary data entry

Four options for purchasing commissary food packs were provided by the jail. Each commissary pack was considered as an independent addition to the weekly seven-day cycle menu and entered into NutritionCalc® Plus and ASA24® as a new profile. Each commissary pack comprised shelf-stable, ready-to-eat foods that contained all items without opportunities for substitution. Based on contents, the commissary packs were categorized as “Dinner Pack”, “Snack Pack”, “Breakfast Pack”, and “Sweets Pack.” The Dinner Pack ($35.00) included 23 entrée items, such as ramen noodles and tuna. The Snack Pack ($26.00) included 23 single-serving savory snack items, such as peanuts and potato chips. The Breakfast Pack ($21.50) included 52 breakfast-type items, such as freeze-dried coffee, sugar packets, and honey buns. The Sweets Pack ($10.00) included eight single-serving sugary snacks, such as cookies and candy. The macronutrient results from each food pack were then individually determined. It was assumed that all food items in the food pack were consumed within a week. To note, each commissary food pack was evaluated in combination with the seven-day cycle menu as well as independently as individuals incarcerated might only consume the food in the commissary food pack if they choose to not eat the food provided by the jail.

### Data evaluation

*Constructing total values and mean values of energy, protein, carbohydrate, total fat, saturated fat.* Mean values for protein, carbohydrate, total fat, and saturated fat in the seven-day cycle menu and for the combination of the menu plus each individual food pack were calculated by summing each macronutrient and dividing by seven. Mean daily calories were calculated by summing calorie content from all meals provided by the seven-day cycle menu and for the combination of the menu plus each individual food pack and dividing by seven. The 2020–2025 Dietary Guidelines for Americans (DGAs) provide healthy dietary patterns guidelines for U.S. adults ages 19 through 59 years, with daily calorie level of patterns ranging from 1,600–3,000 kcal [10]. Available calories were compared to the 2,000 kcal dietary recommendation established in the 2020–2025 DGAs, falling within the range of caloric recommendations for all sedentary adults, and used for general nutrition indicated on a standard nutrition facts label [10].

*Constructing percentage of total calories from grams of added sugars.* ASA24® was used to collect nutrition information and amounts of food groups consumed from the menu and commissary items to determine their added sugars. To analyze the percentage of added sugars in the diet, totals for each day of the seven-day cycle menu and each of the commissary food packs were used. Mean values were calculated for the seven-day cycle menu and the seven-day cycle menu plus the individual commissary food packs. Grams of added sugars were converted to calories, and then divided by 2,000 kcal to generate the percentage of added sugars.

*Constructing percentage of total calories from nutrients for comparison to Acceptable Macronutrient Distribution Range (AMDR).* Using a 2,000 kcal diet standard, the percentage of calories from each macronutrient was compared to AMDR percentages for the studied macronutrients [10]. Grams of protein, carbohydrate, and added sugars were converted to calories (4 kcal/g), and then divided by 2,000 kcal to generate percentages of protein, carbohydrate, and added sugars. Grams of total fats and saturated fats were converted to calories (9 kcal/g) and then divided by 2,000 kcal to generate percentages of total fats and saturated fats. A similar approach was taken to evaluate individual commissary food packs.

*Constructing percentage of total grams of protein and carbohydrate for comparison to Dietary Reference Intakes (DRI)*. The Recommended Dietary Allowance (RDA) for protein consumption for adult men and women is 0.8 g protein/kg body weight/day [24]. Active adults are advised to consume 130 g carbohydrate/day [10]. Grams of protein and carbohydrate provided by the seven-day cycle menu and the four commissary food packs were divided by the RDA, and multiplied by 100 to generate percentage of DRI. The standard male weight of 200 lb (90.9 kg) and standard female weight of 169 lb (76.8 kg) were used to generate the RDA for protein.

*Constructing HEI-2015 scores.* HEI-2015 scores range from 0–100 and comprise adequacy components and moderation components, food groups encouraged for consumption (total fruits, vegetables, greens and beans, whole grains, dairy, total protein foods, seafood, plan proteins, and fatty acids) and food groups recommended for limited consumption (refined grains, sodium, added sugars, and saturated fats), respectively. Estimated HEI-2015 scores indicated how close the jail menu aligns with dietary recommendations, with a higher score representing greater availability of the adequacy component food groups and a lower score representing reduced availability of the moderation component food groups [23]. Data used to generate the HEI-2015 total scores for the seven-day cycle menu and four commissary food packs were computed using the HEI scoring macro in SAS 9.4 (https://epi.grants.cancer.gov/hei/sas-code.html). Mean HEI-2015 was calculated by summing HEI-2015 scores for each day of the seven-day cycle menu and for the combination of the menu plus each individual food pack and dividing by seven.

## Results

### Seven-day cycle menu

**Fig. 1:**
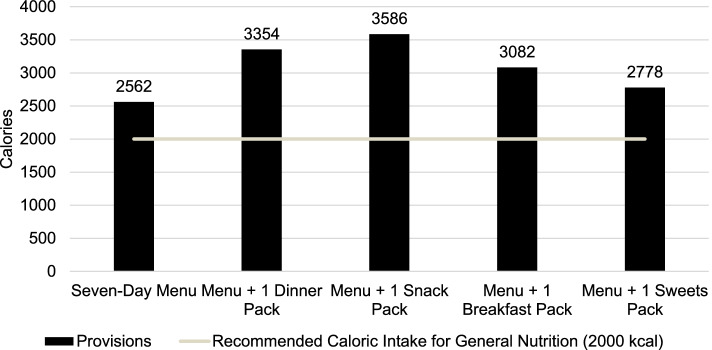
Mean calorie availability of a seven-day cycle menu and commissary food packs at a rural county jail in the Southwestern United States

The mean calorie availability of the seven-day cycle menu [2562 kcal/day (SD = 448)] exceeded recommendations by an excess of 562 kcal (Fig. 1). The mean percentages of calories from protein, carbohydrate, and added sugars for the seven-day cycle menu met AMDR recommendations (Table 2). However, mean percentages of calories from total fat and saturated fat exceeded AMDR recommendations (Table 2).

Daily macronutrient (protein, carbohydrate, total fat) provisions, along with saturated fat and added sugars for the seven-day cycle menu and four commissary food packs are indicated in Table 1. Daily available protein (80.9–126.0 g) was used to determine percentage of calories from protein (ranging between16.2%-25.2% kcal/day over the seven-day cycle), with all days meeting AMDR recommendations for protein (i.e., 10–35% kcal/day). Daily available carbohydrate (262.5–388.6 g) was used to determine percentage of calories from carbohydrate (ranging between 52.5%-66.4% kcal/day over the seven-day cycle), with all days meeting or exceeding AMDR recommendations for carbohydrate (i.e., 45–65% kcal/day). Daily available total fat (79.5–146.7 g) was used to determine percentage of calories from fat (ranging between 35.8%-66% kcal/day), with all days exceeding AMDR recommendations for total fat (i.e., 20–35% kcal/day). Daily available saturated fat (16.7–47.7 g) was used to determine percentage of calories from saturated fat (ranging between 7.5%-21.5% kcal/day), with all days meeting or exceeding AMDR recommendations for saturated fat (i.e., < 10% kcal/day). Daily available added sugars (8.4–14.2 g) was used to determine percentage of calories from added sugars (ranging between 1.7%-2.8% kcal/day), with all days meeting AMDR recommendations for added sugars (i.e., < 10% kcal/day).

**Table 1:** Macronutrient provisions of a seven-day cycle menu and commissary food packs at a rural county jail in the Southwestern United States

Macronutrient	Seven-Day Cycle Menu Provisions							Commissary Food Packs			
	Day 1	Day 2	Day 3	Day 4	Day 5	Day 6	Day 7	Dinner Pack	Snack Pack	Breakfast Pack	Sweets Pack
Protein (grams)	93.5	125.2	80.9	93.6	86.9	84.7	126.0	230.9	152.6	41.9	16.1
Carbohydrates (grams)	282.3	332.0	302.6	303.6	388.6	313.8	262.5	771.4	881.3	597.7	200.1
Total Fats (grams)	112.3	146.7	79.5	118.0	116.7	102.9	143.9	256.4	346.7	134.6	71.4
Saturated Fats (grams)	37.4	47.7	16.7	39.6	33.6	26.6	44.0	90.7	100.6	52.5	16.2
Added Sugars (grams)	8.4	13.8	14.0	10.2	14.2	12.7	12.4	1.4	75.1	52.5	17.1

Percentage DRI for mean protein for men and women exceeded recommendations by 36% and 60%, respectively (Table 2). Percentage DRI for mean carbohydrate for men and women exceeded recommendations by 140%.

**Table 2:** Mean macronutrient provisions, percentage of dietary reference intakes and percentage of daily calories of macronutrients of a seven-day cycle menu and commissary food packs at a rural county jail in the Southwestern United States

Macronutrient	Seven-Day Cycle Menu				Menu + 1 Dinner Pack				Menu + 1 Snack Pack				Menu + 1 Breakfast Pack				Menu + 1 Sweets Pack			
	Mean Provision (g)	% DRI (M)	% DRI (F)	% AMDR	Mean Provision (g)	% DRI (M)	% DRI (F)	% AMDR	Mean Provision (g)	% DRI (M)	% DRI (F)	% AMDR	Mean Provision (g)	% DRI (M)	% DRI (F)	% AMDR	Mean Provision (g)	% DRI (M)	% DRI (F)	% AMDR
Protein	98.7	136	160	19.7	131.7	181	214	26.3	120.5	166	196	24.1	104.7	144	170	20.9	101.0	139	164	20.2
Carbohydrate	312.2	240	240	62.4	422.4	325	325	84.5	438.1	337	337	87.6	397.6	306	306	79.5	340.8	262	262	68.2
Total Fat	117.1	-	-	52.7	153.8	-	-	69.2	166.7	-	-	75.0	136.4	-	-	61.4	127.3	-	-	57.3
Saturated Fat	35.1	-	-	15.8	48.0	-	-	21.6	49.5	-	-	22.3	42.6	-	-	19.2	37.4	-	-	16.8
Added Sugars	12.2	-	-	2.4	12.4	-	-	2.5	23.0	-	-	4.6	19.7	-	-	3.9	14.7	-	-	2.9

*Note*. % Dietary Reference Intake (DRI) for protein refers to the % Recommended Dietary Allowance for protein (0.8 g/kg body weight/day) using the standard male weight of 200 lb (90.9 kg) and standard female weight of 169 lb (76.8 kg). M represents male and F represents female. % DRI for carbohydrate refers to the % Recommended Dietary Allowance for carbohydrate (130 g/day). There is no established DRI for total fats, saturated fats, or added sugars. AMDR % is the percentage of daily calories derived from each macronutrient, using a 2000 kcal dietary standard. The AMDR recommendations include: protein (10–35% kcal/day), carbohydrate (45–65% kcal/day), total fat (20–35% kcal/day), saturated fat (< 10% kcal/day) and added sugars (< 10% kcal/day)

**Fig. 2:**
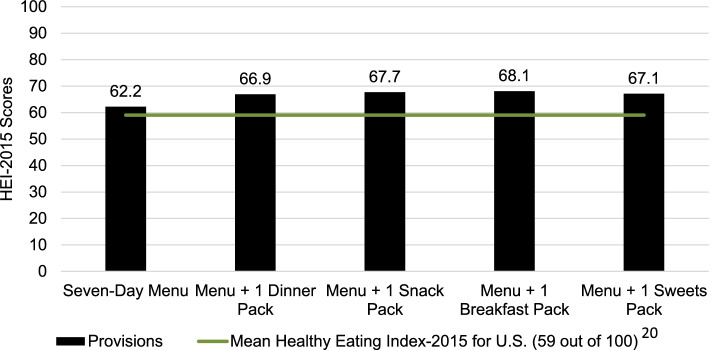
Mean Healthy Eating Index-2015 (HEI-2015) scores of a seven-day cycle menu and commissary food packs at a rural county jail in the Southwestern United States

The mean HEI-2015 total score for the seven-day cycle menu was 62.2 (SD = 9.4) (Fig. 2). Daily HEI-2015 total scores ranged from 49.3–74.5, with the two lowest scores occurring on weekend days (data not shown).

### Commissary food packs

The Dinner Pack provided 6,403 kcal, and if consumed independently from the seven-day cycle menu over a seven-day period, failed to meet the AMDR for protein (6.7% kcal/day), carbohydrate (22.3% kcal/day), and total fat (16.7% kcal/day). Saturated fat (5.8% of kcal/day) and added sugars (0.04% kcal/day) met AMDR recommendations. If consumed with the seven-day cycle menu, daily calories exceeded recommendations, with a possible mean intake of 3,354 kcal/day (Fig. 1). Further, carbohydrate, total fat, and saturated fat exceeded AMDR recommendations, while protein and added sugars met AMDR recommendations (Table 2). The mean HEI-2015 score for the seven-day cycle menu with Dinner Pack was higher than if the seven-day cycle menu was consumed alone (Fig. 2).

The Snack Pack provided 7,196 kcal, and if consumed independently from the seven-day cycle menu over a seven-day period, failed to meet the AMDR for protein (4.3% kcal/day) and carbohydrates (25.0% kcal/day), and met the AMDR for total fat (22.1% kcal/day), saturated fat (6.5% kcal/day), and added sugars (2.1% kcal/day). If consumed with the seven-day cycle menu, daily calories exceeded recommendations, with a possible mean intake of 3,586 kcal/day (Fig. 1). Further, carbohydrate, total fat, and saturated fat exceeded AMDR recommendations, while protein and added sugars met AMDR recommendations (Table 2). The HEI-2015 total score for the seven-day cycle menu with Snack Pack was higher than if the seven-day menu was consumed alone (Fig. 2).

The Breakfast Pack provided 3,639 kcal, and if consumed independently from the seven-day cycle menu over a seven-day period, failed to meet the AMDR for protein (1.2% kcal/day), carbohydrate (16.5% kcal/day), and total fat (8.4% kcal/day) and met the AMDR for saturated fat (3.4% kcal/day) and added sugars (1.5% kcal/day). If consumed with the seven-day cycle menu, daily calories exceeded recommendations, with a possible mean intake of 3082 kcal/day (Fig. 1). Further, carbohydrate, total fat, and saturated fat exceeded AMDR recommendations, while protein and added sugars met AMDR recommendations (Table 2). The HEI-2015 total score for the seven-day cycle menu with Breakfast Pack was higher than if the seven-day menu was consumed alone (Fig. 2).

The Sweets Pack provided 1,521 kcal, and if consumed independently from the seven-day cycle menu over a seven-day period, failed to meet the AMDR for protein (0.46% kcal/day), carbohydrate (5.8% kcal/day), and total fat (4.6% kcal/day), and met the AMDR for saturated fat (1.0% kcal/day) and added sugars (0.49% kcal/day). If consumed with the seven-day cycle menu, daily calories exceeded recommendations, with a possible mean intake of 2,778 kcal/day (Fig. 1). Further, carbohydrate, total fat, and saturated fat exceeded AMDR recommendations, while protein and added sugars met AMDR recommendations (Table 2). The HEI-2015 total score for the seven-day cycle menu with Sweets Pack was higher than if the seven-day menu was consumed alone (Fig. 2).

## Discussion

This study examined the macronutrient distribution, caloric provisions, and dietary quality of a seven-day cycle menu and commissary items from a rural county jail in the southwestern United States. If the seven-day cycle menu was consumed in its entirety, protein, carbohydrate, and added sugars met AMDR recommendations but total fat and saturated fat exceeded recommendations. The DRI for protein was determined using the standard male weight of 200 lb (90.9 kg) and standard female weight of 169 lb (76.8 kg).The current results are consistent with the provision of excess saturated fat in a 28-day cycle menu from a large county jail in Georgia [15]. But compared to the excess sugar provided by a menu from a county detention center in South Carolina [14], added sugars from the current seven-day cycle menu met the AMDR recommendation of < 10% of kcal/day.

Calorie provisions from the seven-day cycle menu exceeded general nutrition recommendations for a 2000 kcal/day diet by 562 kcal. Based upon excess calories available in the seven-day cycle menu, incarceration for a six-month period could result in more than a three-pound weight gain [25]. This timeframe for incarceration is reasonable considering that time spent incarcerated in the county facility from which the menu was evaluated can extend to nearly 3,000 days, with over 25% of individuals re-incarcerated during an 18-month period [6]. The excess saturated fat alone can result in a 932 kcal weekly surplus, resulting in weight gain of over one pound per month. Excess saturated fat and caloric intake may worsen pre-existing cardiovascular disease and other chronic illnesses, as well as contribute to weight gain. These estimates do not consider consumption of commissary food, shelf-stable processed foods that are frequently nutrient-poor and calorie-dense, physical activity level among individuals incarcerated, or different nutritional recommendations based on sex.

Five of the seven days had higher than the national average HEI-2015 score for adults (i.e., 59.0) [26], and the addition of commissary items increased diet quality (> 59 for all four commissary scenarios) indicating overall diet quality is slightly better than that achieved by the average U.S. adult. Previous research points to a Healthy Eating Index-2010 (HEI-2010) score of 74 that would indicate meeting diet quality objectives for Healthy People 2020 [27], whereas a score of 100 meets the 2020–2025 Dietary Guidelines for Americans. Considering that the HEI-2015 score is a density-based measure that considers relative intakes of nutrients based on 1000 kcal, it is possible to see the combination of higher HEI-2015 scores and failure to meet AMDR requirements, such as total fat and saturated fat. HEI-2015 scores on weekend days of the seven-day cycle menu were notably lower than those during the week, with 49.3 on Saturday and 49.5 on Sunday. This may be a result of more meals served on weekends due to greater arrests, no court proceedings, and intermittent “weekend” sentences, while still maintaining a strict budget [15, 28].

The macronutrient distribution of the dinner, snack, and breakfast commissary food packs combined with the seven-day cycle menu met the AMDR recommendations for protein and added sugars but exceeded the DRI for protein and carbohydrate and the AMDR for carbohydrate, total fat, and saturated fat, along with daily calorie recommendations. The macronutrient distribution of the sweets pack with the seven-day cycle menu met the AMDR recommendations for protein, carbohydrate, and added sugars, but exceeded the DRI for protein and carbohydrate and the AMDR for carbohydrate, total fat, and saturated fat, along with daily calorie recommendations. Caloric availability of the menu in addition to a commissary food pack ranged from 2,778–3,556 kcal, exceeding 2020–2025 Dietary Guidelines daily calorie recommendations by 778–1,556 kcal. Carbohydrates in processed foods are often refined, lacking beneficial nutrients such as fiber, vitamins, and minerals. Moreover, highly processed carbohydrates interact with blood sugars differently than minimally processed carbohydrates and may contribute to insulin resistance from overconsumption over an extended period. Excess total fat can also be problematic as the typical Western diet contains a higher ratio of n-6 fatty acids to n-3 fatty acids [29]. Excess n-6 fatty acids are considered pro-inflammatory, contributing to chronic diseases such as cardiovascular disease [30]. Saturated fat contributes to atherosclerosis and increases LDL cholesterol and triglycerides [31]. Excess amounts of these macronutrients in a population already at risk for chronic disease may worsen risk and chronic disease management.

This study has several strengths, including addressing the health of vulnerable populations in rural areas. The nutritional content of commissary foods was assessed in addition to the seven-day cycle menu, addressing a gap in research, and providing a greater understanding of food availability within the jail environment and results that may be generalizable to rural jail populations. Limitations include the lack of objective measurement of food consumption, and the assumption that full portions of the menu would be available for consumption and meals and commissary food packs would be consumed by individuals in their entirety over the course of a week. Because commissary orders are allowed once per week, we feel this is a reasonable assumption to make. Due to seasonality, supply chain issues, and potential limitations in availability of items, the seven-day cycle menu may not be used for the entire year. Additionally, commissary items may change due to availability. One key finding that HEI-2015 scores were higher than those achieved by the average U.S. adult would not be consistent for individuals who fail to consume the seven-day cycle menu in its entirety. Additional limitations include not accounting for the physical activity level of individuals incarcerated, sex differences, and chronic disease, which would affect dietary recommendations. Future research will conduct direct observations of mealtimes in jail settings and measure intake via plate waste to gain a better understanding of consumption. Additionally, interviews and focus groups among individuals incarcerated regarding food consumption, meal preference, commissary habits, and other information regarding healthy eating would contribute to the limited research in this area. The average jail stay is 8–12 days [4, 5], which has limited long-term implications. However, this does not account for recidivism and individuals awaiting trial for longer periods of time. Lastly, the results from the current study are not generalizable to state or federal long-term correctional facilities, such as prisons.

## Conclusions

Although meals provided at a rural southwestern county jail have a higher diet quality compared to the general U.S. population (as measured by the HEI-2015), the seven-day menu and commissary items exceeded calorie and most macronutrient recommendations. While menus served in jails are meant to be nutritionally adequate, they also must consider budget constraints. Possible strategies for maintaining macronutrients within the AMDR, along with diet quality, while lowering available calories should not be directed at removing commissary items, as it is not feasible to substitute fresh items for self-stable foods. Recommendations should focus on improving the seven-day cycle menu by increasing the provision of lower-sodium canned vegetables, no sugar added canned fruits, and fresh and frozen fruits and vegetables by purchasing seasonal produce or by supporting community gardens. To reduce saturated fat intake, facilities should use reduced-fat milk and milk products, bake, roast, or steam foods instead of frying, provide leaner cuts of meats or less beef and pork and opt for poultry, fish, and vegetable proteins, and trim visible fat before cooking.

Food and nutrition play an important role in the management and prevention of chronic disease, especially in vulnerable populations. Improving the health and wellbeing of this population can potentially reduce chronic disease and decrease healthcare costs among incarcerated populations, reducing the burden on public health systems associated with those chronic diseases.

## Supplementary Information

**Additional file 1:** **Supplementary Table 1.** Seven-day Cycle Menu Items that are Missing from NutritionCalc® Plus and the Comparable Item Chosen. 

## Data Availability

Data for the study are not publicly available and will not be shared due to limitations imposed by the southwestern, rural county jail from which they were received.

## Abbreviations

DGA: 2020-2025 Dietary Guidelines for Americans
AMDR: Acceptable Macronutrient Distribution Range
ASA24®: Automated Self-Administered 24-h Dietary Assessment Tool
DRI: Dietary Reference Intake
G: Gram
HEI-2010: Healthy Eating Index-2010
HEI-2015: Healthy Eating Index-2015
IOM: Institute of Medicine
Kcal: Kilocalories
Kg: Kilogram
NY: New York
Lb: Pound
RDAs: Recommended Dietary Allowances
U.S.: United States
USD: United States Dollars
USDA: United States Department of Agriculture
