# Supplementary material for: Nutritional adequacy of meals and commissary items provided to individuals incarcerated in a southwest, rural county jail in the United States
Source: BMC Nutr. 2022 Sep 3;8:96. doi: 10.1186/s40795-022-00593-w (PMC9441029; doi:10.1186/s40795-022-00593-w)
Supplement: Supplementary file 1 — Additional file 1: Supplementary Table 1. Seven-day Cycle Menu Items that are Missing from NutritionCalc® Plus and the Comparable Item Chosen. [file 40795_2022_593_MOESM1_ESM.docx]

| **Supplementary Table 1. Seven-day Cycle Menu Items that are Missing from NutritionCalc® Plus and the Comparable Item Chosen** | |
| --- | --- |
| **Item as listed on 7-day cycle menu** | **Item Chosen from NutritionCalc® Plus** |
| ACI Cookie | cookie, chocolate chip, soft (USDA) |
| Buttered macaroni (8 oz) | 7.5 oz pasta, elbow macaroni, semolina, dry (Golden Grain Mission) + 0.5 oz butter, salted (USDA) |
| Cheese, sliced | cheese product, american, pasteurized processed, sliced (Singles) |
| Chicken corndogs | corn dog, chicken, honey crunchy, frozen (Foster Farms) |
| Chicken jambalaya | jambalaya, chicken & ham (Aramark Campus) |
| Chicken leg quarter | chicken leg quarter, raw, with skin & bone (Foster Farms) |
| Cucumber onion salad (4 oz) | 2 oz cucumber, fresh, without skin, sliced (USDA) + 2 oz onion, white, fresh, chopped (USDA) |
| Dinner loaf RS | meatloaf, baked, with 20% fat beef (USDA) |
| Dry cereal | hot cereal, multigrain, dry, USDA Quaker |
| Fresh baked bread | bread, french, toasted (USDA) |
| Kale salad w/ raspberry vinaigrette (4 oz) | 3.5 oz kale, fresh, chopped (USDA) + 0.5 oz salad dressing, vinaigrette, balsamic (Kraft) |
| Macaroni & cheese | macaroni & cheese, canned (USDA) |
| Margarine reddie | margarine, 80% fat (USDA) |
| Milk, fresh, 2% | milk, 2%, with vitamins A & D (USDA) |
| Pineapple coleslaw (4 oz) | 3 oz coleslaw (USDA: Fast Food) + 1 oz pineapple, fresh, chunks (USDA) |
| Sheet cake | cake, yellow, with vanilla icing, 18 oz (USDA) |
| Shredded potatoes | hash browns, yellow potato, frozen, shreds, with salt (USDA) |
| Spaghetti noodles w/ meat sauce | spaghetti, frozen, with meat sauce (USDA) |
| Spicy ground beef | beef, ground, pan browned, 10% fat (USDA) |
| Super rib | pork back ribs, roasted (USDA) |
| Tossed green salad | salad mix, field greens (Dole) |
| Turkey franks | frank, turkey, classic (Oscar Mayer) |
